# Reassessing Morphological Homologies in the Early-Divergent Angiosperm *Fenerivia* (Annonaceae) Based on Floral Vascular Anatomy: Significance for Interpreting Putative Homeotic Mutations

**DOI:** 10.1371/journal.pone.0081923

**Published:** 2013-12-11

**Authors:** Bine Xue, Richard M. K. Saunders

**Affiliations:** School of Biological Sciences, The University of Hong Kong, Hong Kong, China; University of Kent, United Kingdom

## Abstract

*Fenerivia* species (Annonaceae) are characterized by a prominent flange immediately below the perianth, which has been interpreted as synapomorphic for the genus. The homology of this flange is controversial: previous studies of *Fenerivia heteropetala* (an aberrant species, with 12 perianth parts in three whorls) have suggested that the flange may represent a vestigial calyx resulting from a disruption to the homeotic control of organ identity during floral development. Comparative data on floral vasculature in *Fenerivia capuronii* are presented to elucidate the homology of the flange in other *Fenerivia* species (which possess nine perianth parts in three whorls, typical of most Annonaceae). The flange in *F. capuronii* differs from that in *F. heteropetala* as it is unvascularized. It is nevertheless suggested that the flange is likely to be homologous, and that a homeotic mutation in the *F. heteropetala* lineage resulted in the formation of a vestigial but vascularized calyx that fused with the otherwise unvascularized flange.

## Introduction

The Annonaceae is one of the most species-rich families of early-divergent angiosperms, with 2,292 species recognized in 108 genera [1]. Despite this considerable taxonomic diversity, most species are uniform in their underlying floral structure: the flowers are typically hermaphroditic and protogynous, with a tripartite perianth consisting of a whorl of three sepals and two whorls of three petals that are generally morphologically distinct. Saunders [2] discussed this morphological uniformity in the context of functional convergence, highlighting, for example, the widespread occurrence of pollination chambers formed by the corolla, which are structurally diverse and have evolved independently yet are functionally similar [3]. Saunders’ review [2] of floral evolution in the family attempted to identify similar morphologies in different lineages and hence to identify common explanations; putative mutations to the homeotic control of organ identity during floral development, in particular, were highlighted as potentially having a profound effect on the evolution of floral structure in some lineages. 

Study of floral vascular anatomy provides a wealth of structural information that is invaluable in interpreting evolutionary changes, including the consequences of putative homeotic mutations. Several studies [4–9] have demonstrated that most Annonaceae flowers possess a consistent vascular anatomy, comprising a perianth cortical vascular system (CVS, sensu Deroin [6,10]) in which the sepals are supplied by three basal traces (one median and two lateral traces) and the petals each generally possess a single basal trace: the vascular traces supplying the outer petal whorl are basally fused with the two lateral traces feeding adjacent sepals, whereas the vascular traces supplying the inner petal whorl are basally fused with the median sepal traces.

The Madagascan endemic species *Fenerivia heteropetala* Diels (Annonaceae subfam. Malmeoideae tribe Fenerivieae [1]) was described by Diels [11] based on a peculiar specimen (*H. Perrier de la Bâthie 4942*, P) with an extraordinary floral structure. According to Diels [11], the perianth of *F. heteropetala* consists of three minute sepals (forming a small flange) and 12 petals in three whorls; the outermost corolla whorl consists of three ovate petals that are much broader than the other nine petals, which are linear and arranged in a middle whorl of three and an innermost whorl of six. Other plant morphologists have presented alternative interpretations of the perianth structure in this species, however. Ghesquière [12] regarded the three outermost ovate perianth parts as sepals, and the nine linear perianth parts as two petal whorls, with the inner whorl abnormally duplicated into six petals. Ghesquière [12] did not specifically comment on the homology of the flange (previously interpreted by Diels as a highly reduced calyx), although Deroin [13] suggests that Ghesquière may have considered the flange as an extra-floral nectary. Others, in contrast, have suggested that the flange may merely represent an artefact resulting from specimen dehydration [14]. 

**Figure 1:**
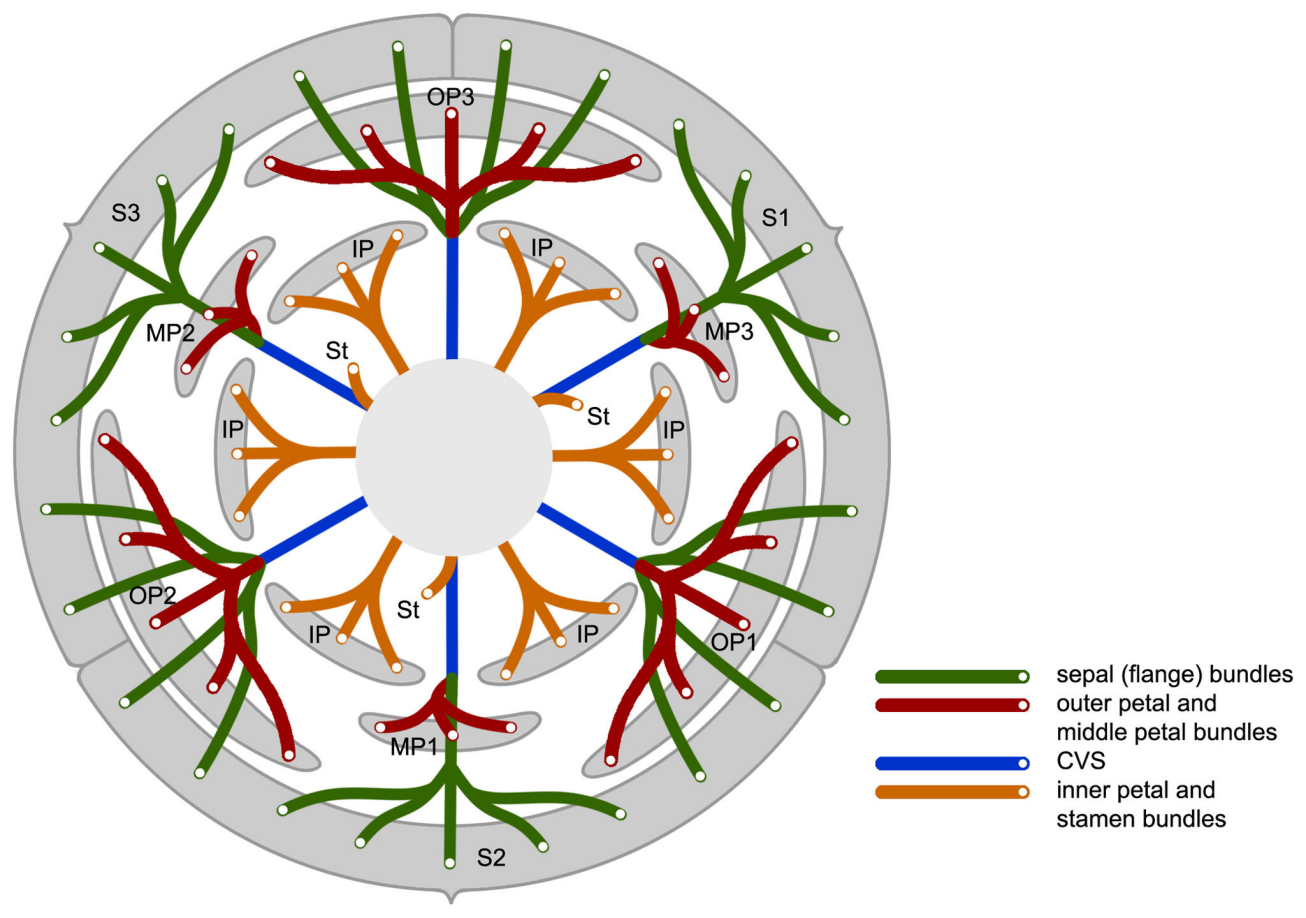
Vascular diagram of *Fenerivia heteropetala* (bundles indicated for perianth and outermost stamen whorl only). Abbreviations, with numbers used to differentiate between organs of the same whorl: S, sepal; OP, outer petal; MP, middle petal; IP, inner petal; CVS, cortical vascular system; St, stamen. Redrawn from Deroin [13].

Deroin [13] undertook an anatomical study of a single floral receptacle from the type collection of *F. heteropetala* and noted that the flange is supported by three clusters of vascular traces (each consisting of one median and two lateral clusters of traces, shown in green in Figure 1); as noted above, this arrangement is typical for the calyx in other Annonaceae, and Deroin therefore regarded the flange in *F. heteropetala* as consisting of reduced sepals, consistent with Diels’ earlier interpretation [11]. The three ovate outer petals (OP 1-3 in Figure 1) alternate with these vestigial sepals and have a vascular supply that is basally fused with the lateral traces in the reduced sepals, forming part of the CVS. The nine linear petals were shown by Deroin to be of two types: three middle petals (MP1-MP3 in Figure 1) with vascular traces basally fused to median calyx traces (also forming part of the CVS), and which therefore resemble the inner petals of other Annonaceae species; and six inner petals (IP in Figure 1), which are supplied by free traces as in the stamens, and are therefore likely to be derived from stamens. Saunders [2] suggested that this may have arisen as a result of a disruption to the homeotic genetic control of organ identity during floral development, with a centrifugal shift in gene expression so that the outermost whorl of six stamens develop as inner petals, the inner petals develop as outer petals, the outer petals develop as sepals, and the sepals fail to develop, resulting in the vestigial calyx flange. 

The genus *Polyalthia* Blume has historically been highly heterogeneous in its delimitation, and has recently been the focus of several phylogenetic studies aiming to reclassify it as a series of strictly monophyletic groups [15–19]. One of these studies [16] has shown that nine *Polyalthia* species from Madagascar form a well-supported clade with *Fenerivia heteropetala*. Although these *Polyalthia* species exhibit the standard number of petals typical of most Annonaceae species (three sepals in one whorl and six petals in two whorls), they also possess a flange at the apex of the pedicel. Saunders et al. [16] regarded this flange as homologous with the structure observed in *F. heteropetala* and hence a conspicuous synapomorphy for the entire clade, and this was cited in support for expanding the taxonomic delimitation of *Fenerivia* to include these nine Madagascan *Polyalthia* species. 

The putative homology of the flange in *F. heteropetala* and its congeners in the newly expanded genus implies a common causal explanation. One hypothesis is therefore that the flange may have originated from the same homeotic mutation and that all *Fenerivia* species are likely to possess identical vascular patterns in the flange. If this is the case, then the two whorls of petals evident in the *Fenerivia* species with six petals (all species except *F. heteropetala*) would not be homologous with the inner and outer whorls of other Annonaceae. The present study therefore aims to investigate floral vascularization in the genus to assess flange homology in the six-petalled *Fenerivia* species, paralleling previous observations in *F. heteropetala* [13].

## Materials and Methods

**Figure 2:**
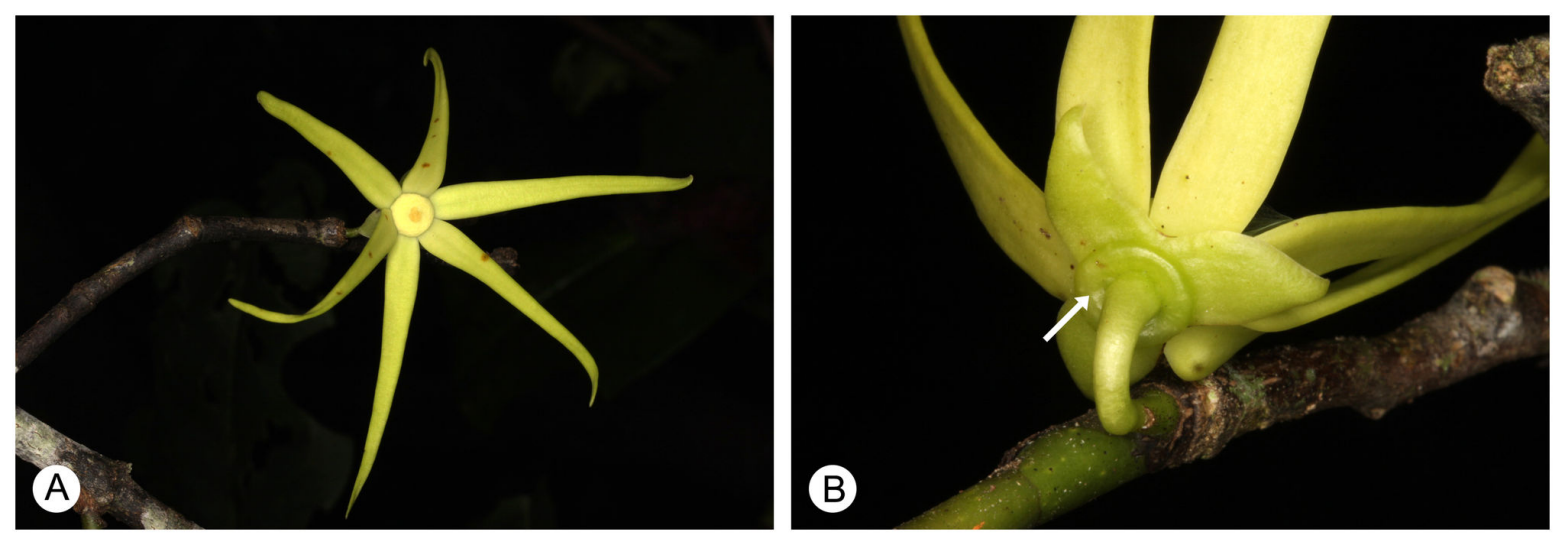
Flower morphology of *Fenerivia capuronii*. (A) Adaxial view of flower, showing the six linear inner petals. (B) Abaxial view of flower, showing the three broad ovate sepals and the flange (arrowed). Reprinted from [16] under a CC BY license, with permission from Lars Chatrou, original copyright 2011.

Two alcohol-preserved flowers of *Fenerivia capuronii* (Cavaco & Keraudren) R.M.K. Saunders (*L.W. Chatrou* et al. *669*, L) were used for serial sectioning and staining. *Fenerivia capuronii* superficially has the standard perianth arrangement for the Annonaceae, with nine perianth parts in three whorls (Figure 2). This species also possesses the distinctive flange (Figure 2B, arrow) that has been regarded as synapomorphic for the entire genus [16].

The flowers of *F. capuronii* were dehydrated using a tertiary-butyl alcohol (TBA) series and embedded in paraffin wax [20]. Serial sections were cut at a thickness of 12 µm using a rotary microtome, and then sequentially mounted onto clean slides using albumin or gelatin. After drying, the slides were stained with Safranin O and Fast Green [20,21] using a Leica ST5020 Multi-stainer, and mounted with DPX (voucher slides deposited in HKU herbarium). Serial sections were photographed using a Leica M205C imaging system, and the pattern of floral vasculature reconstructed by hand. 

Scanning electron microscopy was employed to examine the surface of the flange for the presence of stomata, which is potentially useful in determining organ identity as an extra-floral nectary. As the number of available *F. capuronii* flowers was inadequate, flowers from herbarium sheets of *F. emarginata* (Diels) R.M.K. Saunders (*D. Ravelonarivo* et al. *350*, WAG) and *F. humbertii* (Cavaco & Keraudren) R.M.K. Saunders (*N. Messmer & F. Andriatsiferana NM732*, WAG) were selected as substitutes. Specimens were directly attached to metal stubs using adhesive carbon tabs, sputter-coated with gold/palladium, and viewed using a Hitachi S-3400 VP or Hitachi S4800 FEG scanning electron microscope (SEM) at 5 kV. 

## Results

**Figure 3:**
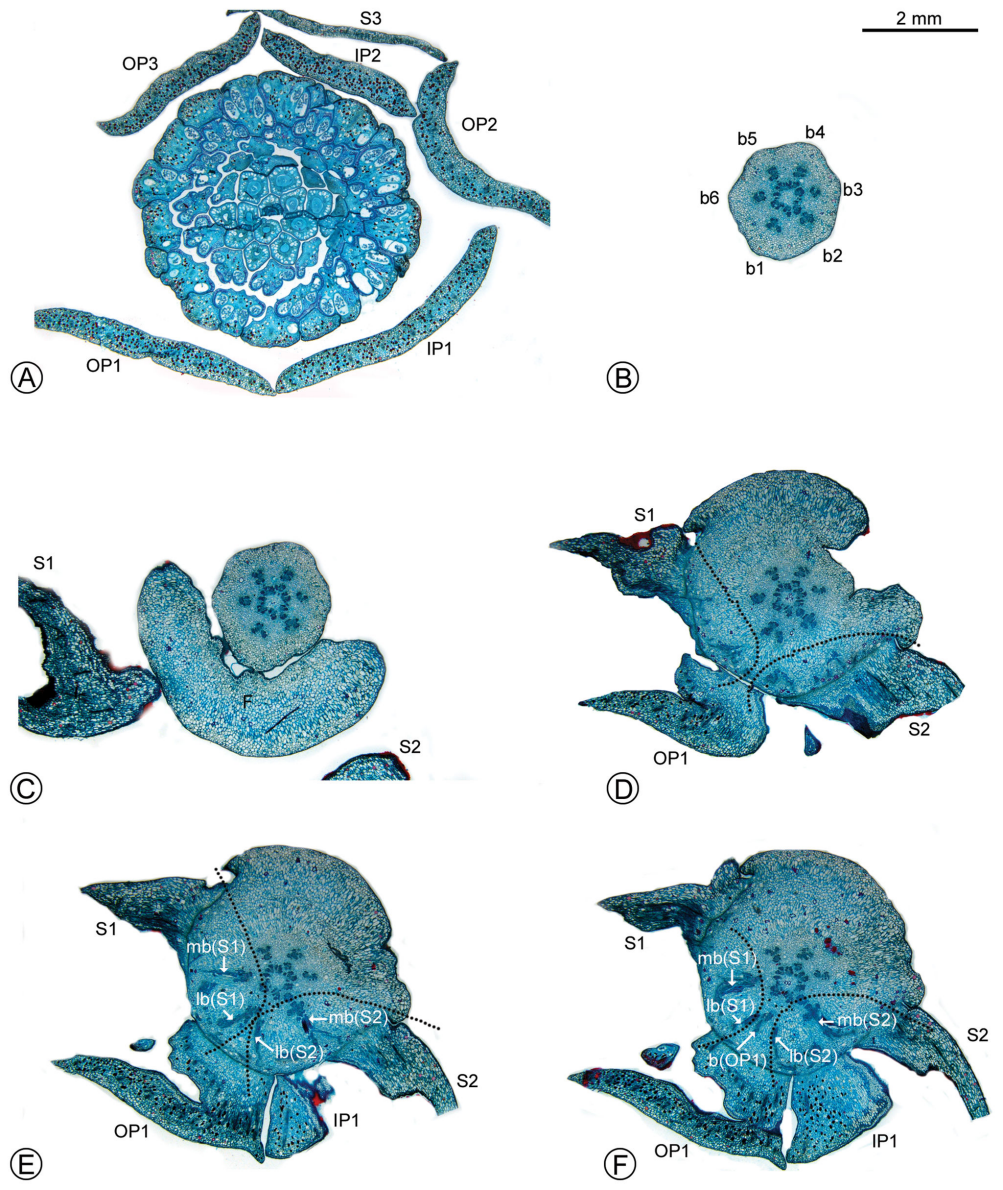
Serial transverse sections through the receptacle of *Fenerivia capuronii*, showing floral vasculature. (B–F in sequence, from base to apex of receptacle.) (A) Section through flower, above receptacle, showing positions of stamens and carpels relative to the sepals, outer petals and inner petals. (B) Base of the receptacle, showing six groups of vascular bundles. (C) Base of the receptacle, showing the flange. (D) Position where the sepals are connected, showing the first appearance of vascular traces. (E, F) Position where the sepals and outer petals are connected, showing their median bundles and lateral bundles. Abbreviations for organs (upper case) and vascular bundles (lower case), with numbers used to differentiate between organs of the same whorl: F, flange; S, sepal; OP, outer petal; IP, inner petal; b, bundle; lb, lateral bundle; mb, median bundle.

**Figure 4:**
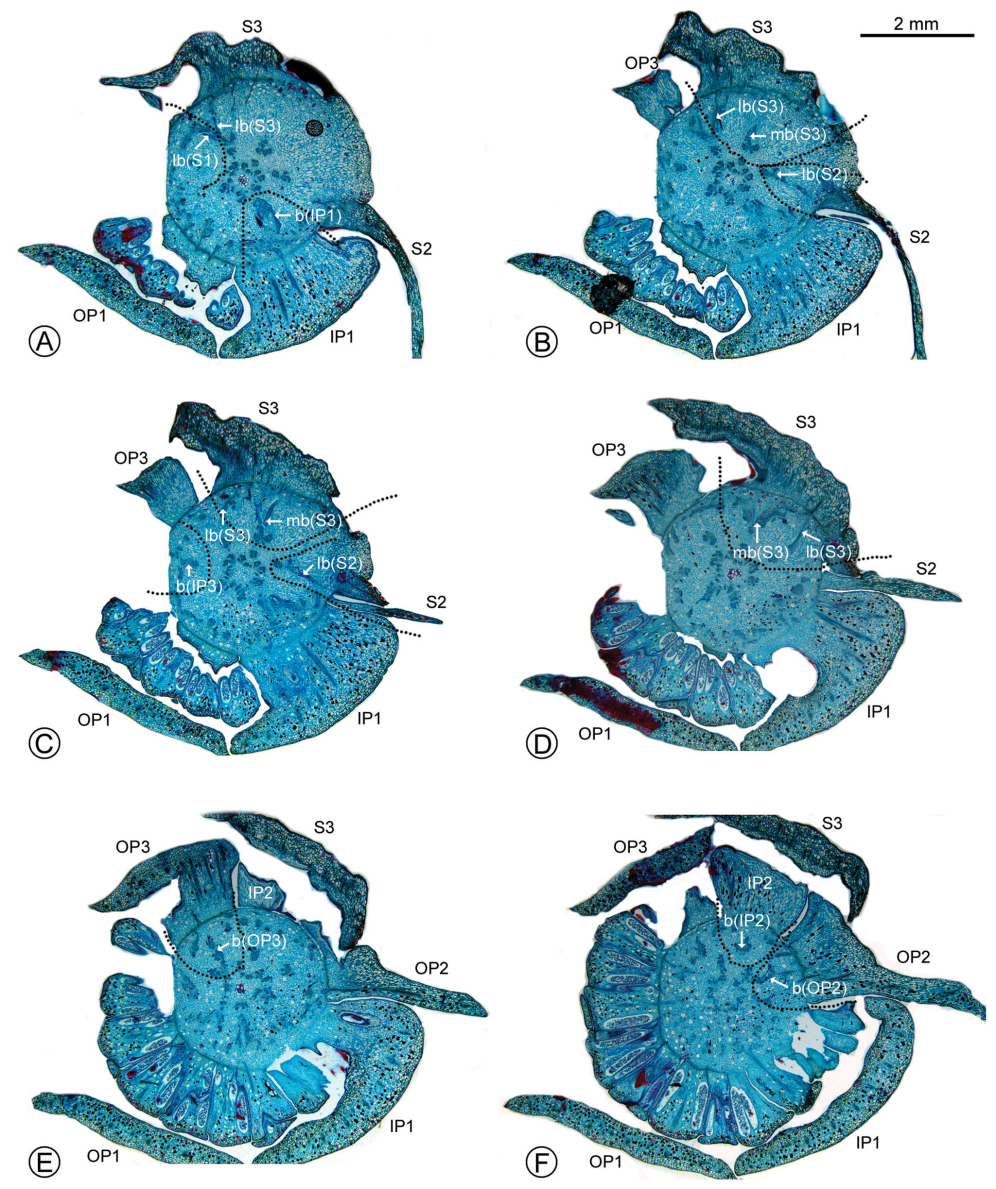
Serial transverse sections through the receptacle of *Fenerivia capuronii*, showing floral vasculature. (A–F in sequence, from base to apex of receptacle.) (A–D) Position where the sepals, outer petals and inner petals are connected, showing the median bundles and lateral bundles of the sepals and bundles leading to the petals. (E) Vascular bundles leading to outer petal 3. (F) Vascular bundles leading to inner petal 2 and outer petal 2. Abbreviations for organs (upper case) and vascular bundles (lower case), with numbers used to differentiate between organs of the same whorl: S, sepal; OP, outer petal; IP, inner petal; b, bundle; lb, lateral bundle; mb, median bundle.

**Figure 5:**
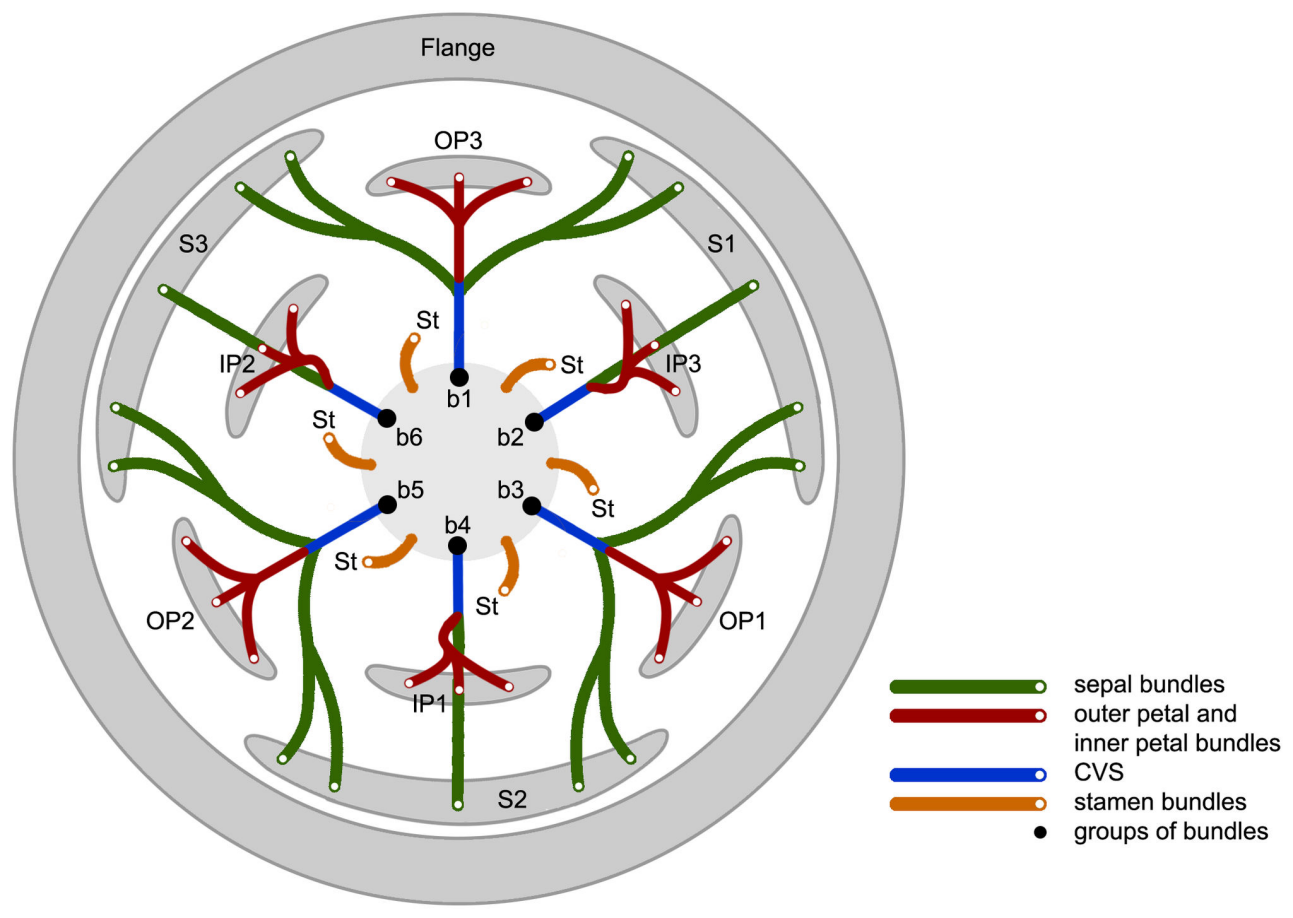
Vascular diagram of *Fenerivia capuronii*. (bundles indicated for perianth and outermost stamen whorl only.) Abbreviations, with numbers used to differentiate between organs of the same whorl: S, sepal; OP, outer petal; IP, inner petal; CVS, cortical vascular system; St, stamen; b, bundle.

Serial sections through the receptacle of *F. capuronii* (Figures 3, 4) reveal the pattern of vascular supply to each perianth organ (presented diagrammatically in Figure 5). The cortical stele in the pedicel consists of six clusters of vascular bundles (labelled b1–6 in Figures 3B, 5). Three of these clusters (b2, b4 and b6 in Figures 3B, 5) connect with the median (but slightly oblique) bundle (mb in Figures 3E, 3F, 4B–D) supplying the three outer perianth parts and the vasculature of the superposed inner perianth parts. The other three groups (b1, b3 and b5 in Figures 3B, 5) connect with the lateral bundles of the adjacent outer perianth organs (lb in Figures 3E, 3F, 4A–D) and the vasculature of the alternate middle perianth parts.

The vascular traces only begin to diverge at the point of attachment of the three broadly ovate outer perianth organs. Each of these organs is supplied by three groups of bundles: two lateral bundles and one median bundle (lb and mb, respectively, in Figures 3E, 3F, 4A–D, 5). The vascular supply to the middle perianth whorl originates from the same source as the lateral bundles of the adjacent outer perianth parts (b in Figures 3F, 4E, 4F, 5). The inner perianth parts are similarly supplied by vascular bundles from the same source as the median bundles of the opposing outer perianth whorl (b in Figures 4A, 4C, 4F, 5). 

**Figure 6:**
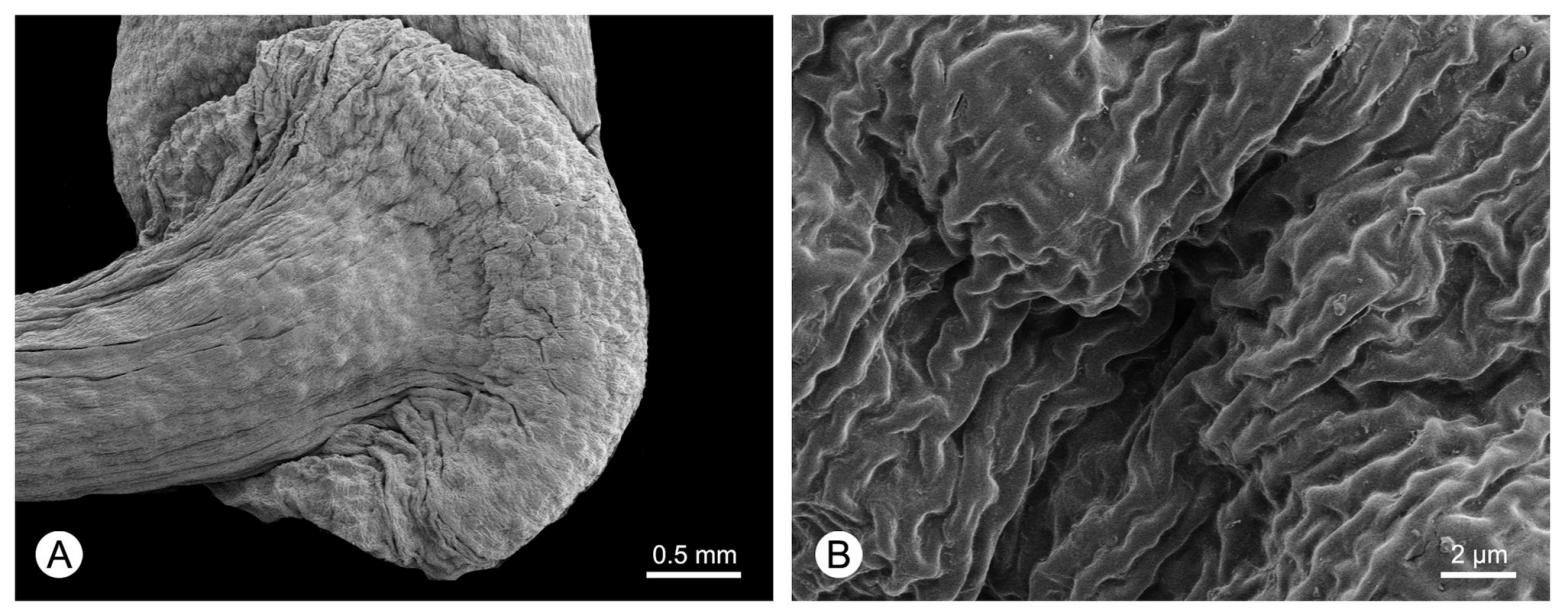
Scanning electron micrographs of the surface of the flange of *Fenerivia humbertii* (*N. Messmer & F. Andriatsiferana NM732*, WAG).

There is no evidence of vasculature in the flange (Figure 3C). There is furthermore no histological evidence that the flange functions as a nectary (Figure 3C), and no stomata were observed on the epidermis (confirmed by SEM studies of two substitute species, *F. emarginata* and *F. humbertii*: Figure 6). 

## Discussion

The vasculature of the middle perianth whorl in *Fenerivia capuronii* is clearly fused to the lateral bundles of the adjacent outer perianth parts, and the vasculature of the inner perianth whorl is similarly fused to the median bundle of each outer perianth part (Figure 5). The vascular traces leading to the various perianth organs are therefore intimately connected, collectively forming a perianth cortical vascular system (CVS, sensu Deroin [6,10]) that conforms with the pattern widely reported in most other Annonaceae flowers [4–9]. The three perianth whorls are accordingly interpreted here as homologues of the calyx and inner and outer corolla whorls observed in other Annonaceae (and are labelled as such in Figures 3–5). The flange in *F. capuronii* is clearly unvascularized (Figure 3C), providing further support for its non-calyx origin.

The floral vasculature and inferred homologies described above for *F. capuronii* contrast strongly with that previously described for *F. heteropetala*: Deroin [13] observed that the flange in *F. heteropetala* is supplied by a pattern of vascular traces that is typical for the annonaceous calyx, and he therefore concluded that the flange was likely to be derived from sepals by reduction. The vascularization of the flange in *F. heteropetala* [13] was subsequently cited as evidence of a probable disruption to the homeotic control of organ identity during floral development [2]. There is clearly no evidence for a homeotic mutation in *F. capuronii*, however, as the two petal whorls of this species are part of the CVS. Both petal whorls are hence inferred to be of bracteal origin, with no evidence of staminal origin.

The flanges in *F. heteropetala* and *F. capuronii* are unlikely to have evolved independently and to be non-homologous. The monophyletic status of the *Fenerivia* clade (including both *F. capuronii* and *F. heteropetala*: [16]) provides a cogent argument in support of this presumed homology, as does the considerable morphological similarity in non-floral characters (e.g., leaf venation) and the narrow and overlapping geographical ranges of the species [14,22,23].

If the homology of the flanges in *F. capuronii* and *F. heteropetala* is accepted, the apparent difference in vascularization of the flanges in the two species could be explained by the fusion of the unvascularized flange and a highly reduced (but fully vascularized) calyx in *F. heteropetala*. Circumstantial support for this is provided by the illustrations previously published by Figures 3D, E in Deroin [13], which show that the lower part of the flange of *F. heteropetala* lacks any vascular traces, and that sepal traces are restricted to the upper region. It is therefore possible that the flange is synapomorphic for the 10 species recognized in *Fenerivia*, and that a homeotic mutation in the *F. heteropetala* lineage [2] resulted in the formation of a vestigial but vascularized calyx that fused with the unvascularized flange. If this explanation is accepted, the flange in *F. heteropetala* consists of two adnate structures, with the lower part homologous with the flange of *F. capuronii*, and the upper part homologous with the calyx of *F. capuronii* and other *Fenerivia* species*.*

It is therefore likely that the disruption to the homeotic control of organ identity during floral development was restricted to *F. heteropetala*, and is not likely to be evident in other species in the genus, which all have the standard number of perianth organs. As only one flowering specimen of *F. heteropetala* has been collected, it is unclear whether the suggested centrifugal homeotic change occurred only in this individual or whether the mutation has been fixed for the species as a whole [16]; additional flowering specimens of *F. heteropetala* need to be collected to clarify this. Investigations of differential homeotic gene expression across the floral meristem are furthermore necessary to unequivocally demonstrate whether a disruption to the homeotic control of organ identity has occurred during floral development.

The function, if any, of the flange in *Fenerivia* is unclear. There is no support for the suggestion (apparently attributed to Ghesquière by Deroin [13]), that the flange may function as an extra-floral nectary: there is no evidence of nectar vascularization in the parenchyma (Figure 3C), which is indicative of structural nectaries [24,25]; nor is there any evidence of stomata or glandular trichomes on the epidermis (Figure 6). The hypothesis that the flange may function as an extra-floral nectary is difficult to refute, however, since extra-floral nectaries can be unvascularized and lack the anatomy typical of nectaries [25]. Unequivocal conclusions on the function of the flange inevitably require field observations of nectar secretion, and histochemical studies to detect the presence of nectariferous compounds (including lipids, polysaccharides, proteins and phenols: [25]). 
